# Changes in Cerebral Blood Flow during an Alteration in Glycemic State in a Large Non-human Primate (*Papio hamadryas* sp.)

**DOI:** 10.3389/fnins.2017.00049

**Published:** 2017-02-14

**Authors:** Peter Kochunov, Hsiao-Ying Wey, Peter T. Fox, Jack L. Lancaster, Michael D. Davis, Danny J. J. Wang, Ai-Ling Lin, Raul A. Bastarrachea, Marcia C. R. Andrade, Vicki Mattern, Patrice Frost, Paul B. Higgins, Anthony G. Comuzzie, Venkata S. Voruganti

**Affiliations:** ^1^Maryland Psychiatric Research Center, University of Maryland School of MedicineBaltimore, MA, USA; ^2^Research Imaging Institute, University of Texas Health Science Center at San AntonioSan Antonio, TX, USA; ^3^Southwest National Primate Research CenterSan Antonio, TX, USA; ^4^Department of Radiology, Athinoula A. Martinos Center for Biomedical Imaging, Massachusetts General Hospital, Harvard Medical SchoolCharlestown, MA, USA; ^5^Ahmanson-Lovelace Brain Mapping Center, University of California at Los AngelesLos Angeles, CA, USA; ^6^Mark and Mary Stevens Neuroimaging and Informatics Institute, Keck School of Medicine, University of Southern CaliforniaLos Angeles, CA, USA; ^7^Department of Genetics, Texas Biomedical Research InstituteSan Antonio, TX, USA; ^8^Center for Laboratory Animal Breeding, Oswaldo Cruz FoundationRio de Janeiro, Brazil; ^9^Department of Nutrition and UNC Nutrition Research Institute, University of North Carolina at Chapel HillKannapolis, NC, USA

**Keywords:** hyperglycemic challenge, cerebral blood flow, perfusion imaging, resting state network, default state network, arterial spin labeling

## Abstract

Changes in cerebral blood flow (CBF) during a hyperglycemic challenge were mapped, using perfusion-weighted MRI, in a group of non-human primates. Seven female baboons were fasted for 16 h prior to 1-h imaging experiment, performed under general anesthesia, that consisted of a 20-min baseline, followed by a bolus infusion of glucose (500 mg/kg). CBF maps were collected every 7 s and blood glucose and insulin levels were sampled at regular intervals. Blood glucose levels rose from 51.3 ± 10.9 to 203.9 ± 38.9 mg/dL and declined to 133.4 ± 22.0 mg/dL, at the end of the experiment. Regional CBF changes consisted of four clusters: cerebral cortex, thalamus, hypothalamus, and mesencephalon. Increases in the hypothalamic blood flow occurred concurrently with the regulatory response to systemic glucose change, whereas CBF declined for other clusters. The return to baseline of hypothalamic blood flow was observed while CBF was still increasing in other brain regions. The spatial pattern of extra-hypothalamic CBF changes was correlated with the patterns of several cerebral networks including the default mode network. These findings suggest that hypothalamic blood flow response to systemic glucose levels can potentially be explained by regulatory activity. The response of extra-hypothalamic clusters followed a different time course and its spatial pattern resembled that of the default-mode network.

## Introduction

Recent evidence suggest that a dysregulation in the central nervous system (CNS)'s control over energy intake, storage, and expenditure contributes to the development of many common metabolic disorders including type 2 diabetes mellitus, insulin resistance, and hyperlipidemia (Jordan et al., 2010). The exact mechanism of CNS dysregulation in the development of these diseases is still unknown. The CNS processes information about the body's nutritional state and acts as the main regulatory center to ensure the maintenance of energy homeostasis (Jordan et al., 2010). There is an urgent requirement for biologically relevant models to explain the role of cerebral dysregulation in metabolic disorders; non-human primate (NHP) models have potential for this purpose. NHP models are essential translational models for the study of dysmetabolic conditions and the role of the CNS therein (Cabrera et al., 2006; Pound et al., 2014). It is recognized that small animal models are limited for advancing our understanding of the important neuro-regulatory mechanisms that are key drivers of metabolic health in humans (Burcelin, 2010). For example, the contribution of brain insulin signaling to hepatic glucose production in mice and rats differs markedly from its contribution in larger animals and consequently, rodent models offer only very limited translational value for studying hepatic glucose production: a key driver of elevated plasma glucose in type 2 diabetes (Burcelin, 2010). Hence, NHPs provide critical intermediary models, both for bridging rodent findings to the likelihood of clinical relevance and for identifying novel mechanisms operational only in the higher species. A large body-size NHP, such as the baboon, that enables highly controlled and detailed study at the whole body systems level is critical for advancing our understanding of the CNS's role in metabolic health and disease (Higgins et al., 2016). While the baboon is an established general preclinical model for obesity, cardiovascular disease, and type 2 diabetes (Chavez et al., 2008; Guardado-Mendoza et al., 2009); techniques and methodologies that enable the incorporation of trenchant CNS assessments into studies using this model are currently limited. Advanced brain imaging approaches that are deployable during whole body metabolic studies in this model are particularly lacking.

The rationale of this study, thus, is to aid the development of NHP model of cerebral regulation of metabolic function by combining advanced functional neuroimaging technique with a commonly used metabolic challenge to replicate previous findings in humans. Neuroimaging studies in humans have demonstrated that brain's response to hyperglycemic challenge can serve as a potential biomarker of the severity of cerebral dysregulation observed in diverse metabolic disorders such as obesity, type 2 diabetes mellitus, and hypoglycemic unawareness (Matsuda et al., 1999; Choi et al., 2003; Criego et al., 2005). Specifically, an inhibited and/or time-delayed brain's response to infusion of nutrients under a fasting glycemia was observed in patients with metabolic disorder and reversal of this response was suggested to be a potential treatment target for pharmacological treatments (Matsuda et al., 1999). We compared the trends observed in baboons, under general anesthesia, to the results of similar studies in humans to help the development of NHP models of dysregulation of cerebral control in metabolic disorders.

Many earlier fMRI studies of CNS responses to hyperglycemic challenge were methodologically limited to a single slice image to achieve the necessary temporal resolution (Matsuda et al., 1999; Liu et al., 2000). The follow up whole-brain PET studies have helped to clarify the physiologic meaning of these findings by demonstrating significant changes in the hypothalamic and extra-hypothalamic cerebral blood flow (CBF) following the glucose infusion (Cranston et al., 2001; Teh et al., 2010). The magnitude and timing of the hypothalamic CBF changes were shown to be correlated with subject's body mass index and fasting levels of glucose and insulin (Matsuda et al., 1999; Liu et al., 2000). The physiological meaning of the extra-hypothalamic CBF changes was more difficult to interpret with many studies leaving it unexplained (Cranston et al., 2001; Teh et al., 2010). Recent improvements in the MRI technology have enabled for time-resolved, non-invasive measurements of CBF throughout the brain using arterial spin labeling (ASL; Detre and Alsop, 1999). A study by Page and colleagues used ASL-fMRI to show that even minor (<10 mg/dL) alterations in glycemia lead to increases in the hypothalamic CBF (Page et al., 2009). This study also observed significant CBF changes in the extra-hypothalamic regions but the gap of 30 min between measurements prevented them from reporting on the temporal relationship between the hypothalamic and extra-hypothalamic CBF changes (Page et al., 2009).

Our pilot study was designed to better characterize hypothalamic and extra-hypothalamic CBF trends during a hyperglycemic challenge. Toward this goal, we combined the ASL-fMRI with peripheral sampling of glucose, insulin and C-peptide to map the transition from fasting glycemia to hyperglycemia. Our aims were to study the hypothalamic involvement in metabolic regulation and to attempt to explain CBF changes in the extra-hypothalamic regions. We performed this study in a large NHP, the baboon (*Papio hamadryas* Sp.). We are developing the baboon as a preclinical animal model to study the physiology and genetics of common human metabolic disorders including insulin resistance, diabetes, obesity, and dyslipidemia (Guardado-Mendoza et al., 2009). Recently, we successfully and safely induced diabetes in conscious baboons through a single dose of pharmaceutical-grade steptozotocin (Chen et al., 2014; Frost et al., 2015). A comprehensive characterization of hypothalamic and extra-hypothalamic CBF will help us better understand metabolic regulation in both forms of diabetic (I and II) conditions. From a neuroimaging perspective, baboons offer the advantage of having among the largest brains of commonly studied laboratory primate (Kochunov et al., 2009). Furthermore, our previous neuroimaging studies in baboons have demonstrated that the baboon model offers clinically relevant structural, functional, physiological, and metabolic information about brain structure, function, development, and genetic variability (Kochunov et al., 2009; Wey et al., 2010).

## Materials and methods

### Animal subjects and experimental protocol

Seven adult (mean age = 9.17 ± 1.2 years [range: 8.4–11.7 years]) female baboons (*P. hamadryas* Sp.) were selected from a large, breeding colony maintained by the Southwest National Primate Research Center (SNPRC). To reduce potential gender-related differences in regional glucose metabolism, we selected female animals in this study. The average body weight was 17.9 ± 3.5 kg (range 15.4–25.4 kg). All animals had a stable weight pattern (<3% change over the last 12 month) with normal euglycemic blood glucose values ([Glc] = 89 ± 9 mg/dL) on entry to the study. Animal handling and anesthesia protocols were optimized for fMRI and are described elsewhere (Kochunov et al., 2009). Animals were fasted for 16 h, with full access to water, before being transported from the SNPRC to the animal preparation area at the Research Imaging Institute (RII) at the University of Texas Health Science Center at San Antonio (UTHSCSA). Each animal was sedated with an intramuscular injection of s-ketamine 10 mg/kg (KetaVed., Phoenix Scientific, St. Joseph, Missouri), intubated with an MR-compatible tracheal tube and 18 gauge catheters were inserted into the left and right saphenous veins. Animals were then moved to the MRI room where anesthesia was induced and maintained by mechanical ventilation, at the rate of 10 respiration/minute, with 2% Isoflurane, Animal's heart rate, end-tidal CO_2_ concentrations and core body temperature were monitored using MRI compatible equipment from 15 min prior to imaging and throughout the imaging experiment. Each 60 min long fMRI session consisted of 20 min of baseline imaging followed by a bolus injection of glucose (dextrose, 50%) calculated at 500 mg/kg of body weight into the left saphenous vein. All animal protocols were reviewed and approved by the Institutional Animal Care and Use Committee of Texas Biomedical Research Institute.

### Peripheral measurements

Blood was drawn from the right saphenous vein at 5 min intervals for glucose measurements in all animals. In addition, 5 ml blood draws were performed at 0, 10, 20, 22.5, 27.5, 35, 45, and 55 min to ascertain insulin and C-peptide plasma concentrations in five of the seven animals. Whole blood glucose was measured with a glucometer (Accu-chek AVIVA, Roche Diagnostics). We confirmed its accuracy ([Glc]plasma = 1.12 × [Glc]blood + 4.1; *r*^2^ = 0.99; *p* < 10-4) by analyzing 10 random glucose measurements, in the first two animals, using the Alfa Wasserman ACE clinical chemistry instrument (West Caldwell., NJ). Plasma insulin and C-peptide were measured by immunoassay using the Immulite Analyzer (Diagnostic Products Corporation, Los Angeles, CA).

### Imaging

Measurement of cerebral perfusion was performed using a pseudo-continuous ASL EPI sequence with the following parameters: TR/TE = 3,500/16 ms, labeling duration = 2.1 s, post-labeled delay of 700 ms, 15 contiguous slices with 3 mm slice thickness, matrix = 64 by 64, FOV = 12.8 × 12.8 cm (2 × 2 × 3 mm resolution), labeling gradient of 0.6 G/cm with details presented elsewhere (Wey et al., 2010). Alternating labeled and non-labeled images were acquired in pairs. Co-registered., high-resolution, 3D structural images were collected using an optimized structural protocol described elsewhere (Kochunov and Duff Davis, 2009).

At the end of the study, the mean transit time (MTT) between the site of injection and cerebrum was measured using a bolus of an exogenous agent (gadodiamide). A human adult dose (0.2 ml/kg) of gadodiamide (OMNISCAN, GE Healthcare, USA) was administered to left saphenous vein. A susceptibility (T2^*^) sensitive EPI sequence with TR = 1 s was used to image the first pass of the contrast agent through the cerebral circulation. In the concentration used for the bolus, gadodiamide is capable of significantly (~5- to 10-fold) shortening the relaxation times of blood with blood's signal intensity reduced in linear proportion to the concentration of the contrast agent.

### Image preprocessing

Images were pre-processed using the FMRIB software library (www.fmrib.ox.ac.uk/fsl/). The pre-processing steps included: correction for head motion artifacts, removal of non-brain tissue and calculation of perfusion weighted images using a two compartment model as described elsewhere (Wey et al., 2010). All images were spatially registered to a population-average baboon brain atlas as described in a prior manuscript (Kochunov and Duff Davis, 2009).

### Analysis of CBF trends

**Figure 1:**
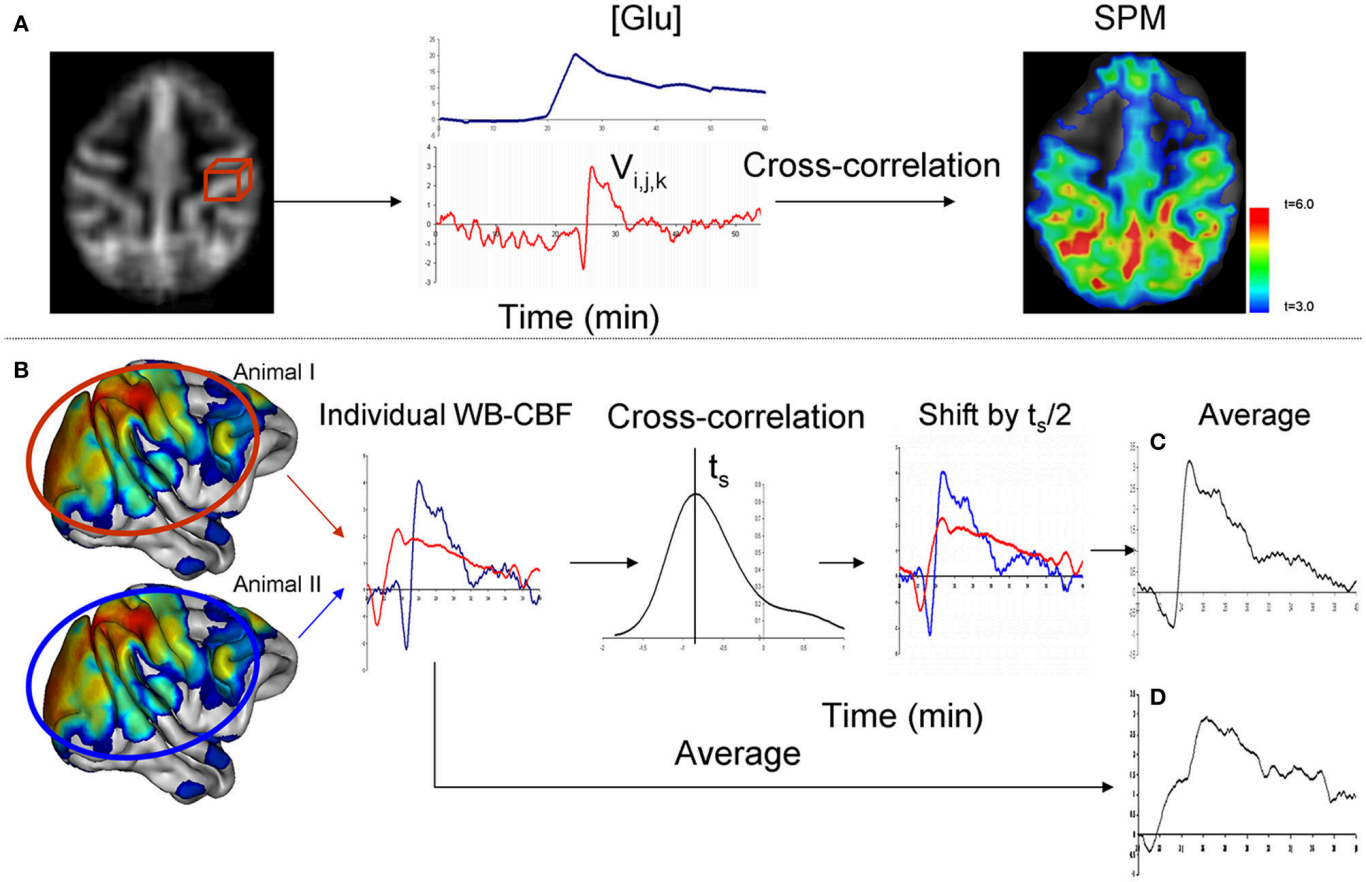
**Data analysis was performed in two steps**. During the first step **(A)**, cerebral area with CBF trends that followed the trends of blood glucose levels ([Glc]) were identified using cross-correlation analysis. During the second step **(B)**, time-shift analysis was performed to calculate the average, across all significant voxels, wave-form and the time of onset for individual animals. Cross-correlation is used to calculate the time (ts) which maximizes the correlation-coefficient between the CBF trends among the individual animals. Correction for individual differences in times provided for a clarified average CBF trend when compared to a simple averaging **(C,D)**.

A data-driven analysis approach was used to identify brain regions, whose CBF were affected by glucose infusion. This approach, developed to study the effects of pharmacological agents on brain function (Salmeron and Stein, 2002), uses the agent's pharmacokinetic time-activity curve as a parametric statistical predictor of cerebral activity. The agent's time-activity curve is assessed by peripheral measurements of its concentration. We use a statistical cross-correlation analysis to identify brain regions where voxel-wise CBF time-activity curve was significantly correlated with temporal profile of the peripheral CBF measurements. The cross-correlation analysis accommodates for potential timing delay between peripheral and cerebral circulation as well as for the inter subject differences in the timing of cerebral activity. We chose the blood glucose level as the predictor variable because glucose level measurements require collection of only small amounts of blood (<0.1 ml compared to 5 ml for measurements of regulatory hormones) and therefore was performed more frequently (every 5 min) for improved temporal sampling frequency. The voxel-wise, cross-correlation analysis was performed using the FSL-FEAT general linear model tool (Smith et al., 2004). The blood glucose temporal trends were treated as the contrast. According to the guidelines given in the FSL-FEAT manual, the CBF and glucose concentration data were normalized by converting to *z*-scores. This was accomplished by subtracting the average baseline values and dividing the result by the baseline's standard deviation. Cross-correlation identified the maximum Pearson correlations between blood glucose trends and the voxel-wise perfusion and for the individual animals (Figure 1). The result of this analysis was the statistical parametric map (SPM) of the voxel-wise statistical significance, expressed as t-statistics. This map was thresholded using the Gaussian random field theory at the significance level of *t* = 3.0 and cluster-wise significance of *p* = 0.01 (Figure 1A).

### Comparison with the spatial pattern of resting state cerebral networks

We interpreted the regional changes in CBF trend following glucose uptake by performing a similarity analysis between the spatial patterns of the glucose-related CBF changes and those of 20 resting state networks measured in seven unrelated, adult female baboons (Wey et al., 2011). The animal handling protocol, maintenance of the anesthesia and mechanical ventilation frequency (10 respirations/min) were the same with an exception that vecuronium bromide (0.1 mg/kg) was used to facilitate endotracheal intubation (Wey et al., 2011). Resting-state fMRI data were collected with high resolution (1 × 1 × 1.9 mm) gradient echo EPI sequence (TR/TE = 3000/30 ms). The spatial patterns of resting state networks were calculated using the FMRIB Software Library (FSL) MELODIC using default settings. This analysis produced 20 temporal-concatenated, independent component (Smith et al., 2004). Pre-processing steps included: motion correction, brain extraction, temporal band-pass filtering (0.01–0.08 Hz), and spatial smoothing (Gaussian kernel, FWHM = 5 mm). All images were registered to a high-resolution baboon brain template and concatenated in time (Kochunov et al., 2009). The 20 components represented the main modes of coactivation across all images. These components included the executive networks, the visual networks, the default mode network, and the sensory-motor network that were identified through visual inspection and confirmed with spatial correlation analysis with the human data (Kelly et al., 2010).

We calculated spatial correlation coefficients between the SPM of the association between temporal glucose and CBF trends using FSL's spatial correlation utility (fslcc) and estimated significance using a two tailed, one-sample *t*-test (Kelly et al., 2010). The level of statistical significance was set at *p* ≤ 0.001 to reduce the probability of Type 1 errors associated with multiple (*N* = 20) comparisons.

### Analysis of temporal differences among regional CBF trends

Next, we analyzed the temporal differences among the regional CBF trends. At the design of the experiment, we expected that individual animals may show temporal differences due to both methodological such as the timing of injection of the bolus contrast and physiological factors such as individual metabolic responses to glucose bolus. First, a cross-correlation analysis was used to correct for intersubject differences in the whole-brain CBF trends. This correction was necessary as demonstrated by Figure 1B. The average CBF curve can be distorted by the individual timing differences if simple averaging is used. The average CBF curve can be restored by shifting individual curves. This analysis was performed by calculating the average CBF trend for all significant voxels. Cross-correlations were then calculated for the average CBF time trend for each animals and the rest of the group. This analysis estimated the shift in time (ts) that maximizes the overlap in the average CBF trends among animals (Figure 1B) and calculated the average temporal CBF curve by correcting for individual temporal differences (Figures 1C,D).

Following this correction, we analyzed the temporal relationship among the CBF trends for four region whose CBF trends showed a significant association with blood glucose levels: cortex, thalamus, hypothalamus, and mesencephalon. These regions were manually segmented by overlaying the average SPM on the model of population-average cortical surface model derived from anatomical data of about 200 baboons (Kochunov and Duff Davis, 2009). The potential co-linearity of the time trends among regions was removed using a data reduction technique—factor analysis. This analysis was performed for the 25 min long window of data that started 5 min prior to glucose infusion. Factor analysis used the principal components analysis (PCA) to obtain linear composites of time trends for different regions of interest with varimax rotation used to orthogonalize the eigenvectors. Results of factor analysis yielded factor loadings (correlations between a time-trends and a factor) and a standardized *Z*-score for each factor.

All images were registered to a high-resolution baboon brain template and concatenated in time (Kochunov and Duff Davis, 2009).

## Results

**Figure 2:**
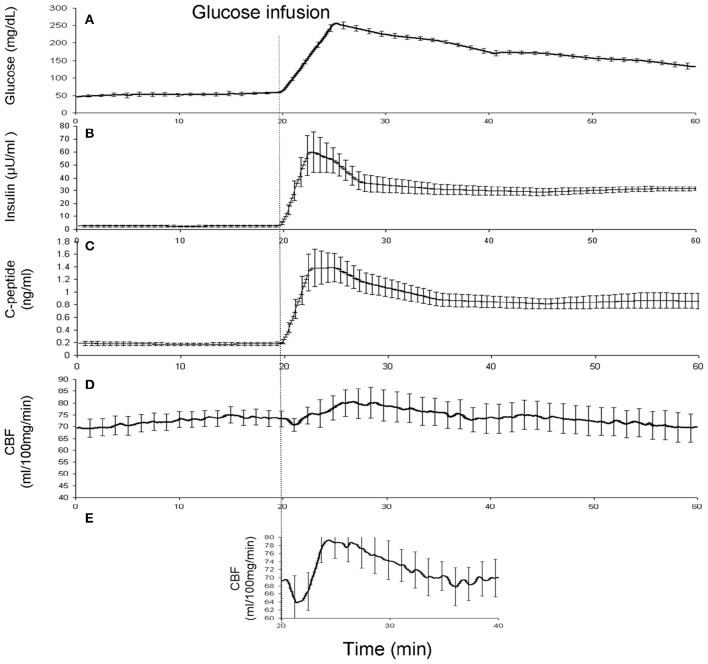
**(A)** Blood glucose concentrations were measured every 5 min and then interpolated between time points and averaged for all animals. **(B)** The plasma Insulin^*^ (middle) and **(C)** C-peptide^*^ concentrations were measured at 0, 10, 20, 22.5, 27.5, 35, 45, and 55 min and then interpolated between time points and averaged for all animals. The whole-brain average CBF curve **(D)** and the average CBF calculated for significant voxels after correction for timing differences **(E)**. ^*^These data were available for 5 out of 7 animals.

The state of fasting glycemia was confirmed by measuring stable baseline blood glucose and plasma levels of insulin and C-peptide (51.3 ± 10.9 mg/dL; 2.6 ± 1.4 μU/ml, and 0.18 ± 0.06 ng/ml, respectively; Figure 2). Infusion of glucose produced short (~20 min) lasting hyperglycemia (peak value = 203.9 ± 38.9 mg/dL) and elevations in insulin and C-peptide levels (Figure 2). During the last 20 min, the blood glucose levels and plasma levels of insulin and C-peptide returned to normal post-meal values (134.4 ± 22.0 mg/dL, 30.6 ± 3.2 μU/ml, and 0.88 ± 0.20 ng/ml, respectively). The whole-brain (WB)-CBF values increased from the average baseline value of 72.4 ± 2.8–76.3 ± 3.2 mg × 100 gm^−1^×min^−1^ (observed at 4.2 min after glucose infusion) and remained elevated for ~15 min (average CBF = 75.1 ± 2.5 mg × 100 gm^−1^×min^−1^; Figure 2). About 35% of the brain voxels showed a statistically-significant (*t* > 3.0; *p* < 0.01) correlation with the glucose concentration trend. The average CBF trend from these regions was corrected for the individual differences in the times of onset by time-shifting the CBF trends. This step clarified the average wave-shape of the whole-brain CBF. The average CBF trend for these regions showed a dip-rise-overshoot trend, with the dip (CBF = 65.7 mg × 100 gm^−1^×min^−1^) occurring at 2.2 min and preceding the peak CBF values (CBF = 79.3 mg × 100 gm^−1^×min^−1^), by about 3 min (Figure 2). During the remaining 20 min the glucose, insulin and C-peptide levels returned to typical post-meal values and the WB-CBF returned to its base-line value (73.2 ± 2.9 mg × 100 gm^−1^×min^−1^).

**Figure 3:**
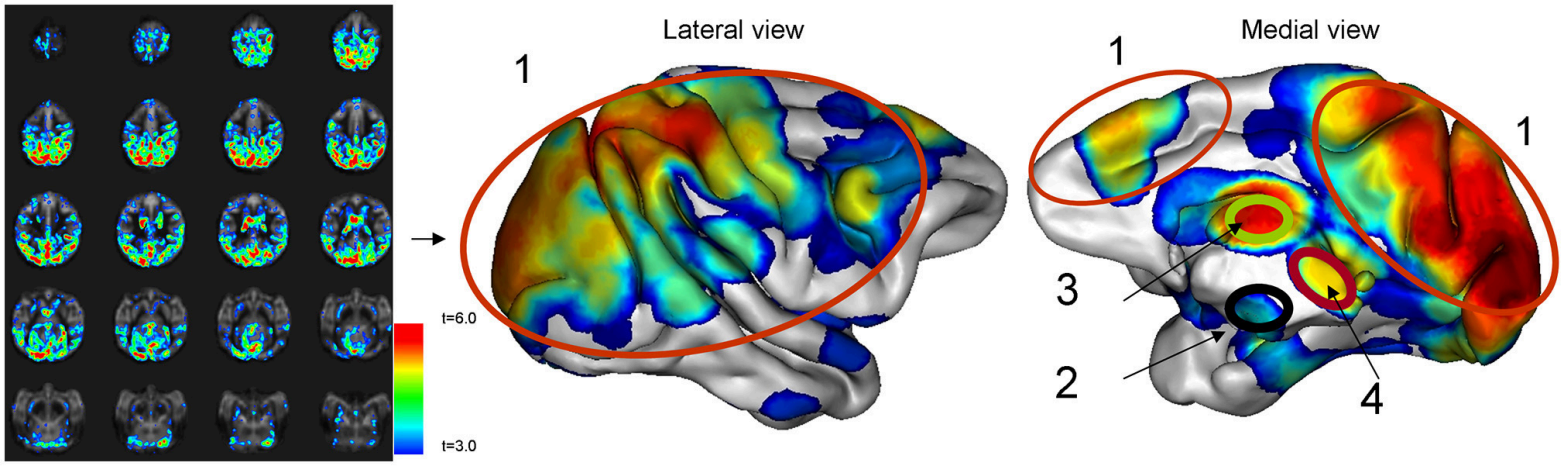
**The whole-brain average CBF curve and the average CBF calculated for significant voxels after correction for timing differences**. The average statistical parametric map of CBF changes due to glucose infusion was rendered on a 3-D cerebral surface.

**Figure 4:**
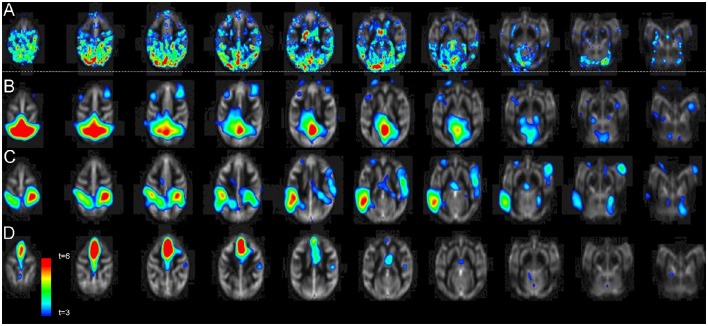
**The average statistical parametric map of CBF changes (A)** Pearson cross-correlation coefficients between glucose infusion and CBF time trends. The pattern **(A)** was significantly correlated with the pattern of default-mode **(B)**, motor-and-sensory **(C)**, and the executive control **(D)** networks (*r* = 0.51, *r* = 0.29, and 0.25, respectively; *p* ≤ 0.0001).

Regional pattern of statistically significant CBF change consisted of four regions: cortex, hypothalamus, thalamus, and mesencephalon (Figure 3). The spatial cross-correlation analysis between this pattern (Figure 4A) and the spatial patterns of 20 resting-state networks was performed to study its overlap with regionally specific patterns of activity during resting state (Figures 4B–D). The highest spatial correlation (*r* = 0.51, *p* = 10^−6^) was observed for the default mode network (Figure 4B) with both images showing similar spatial patterns that included superior partial lobe and the pre-frontal and cingulate areas. Significant spatial correlation were also observed for the motor-and-sensory (Figure 4C) networks (*r* = 0.29 and 0.25; respectively, *p* = 0.0001).

**Figure 5:**
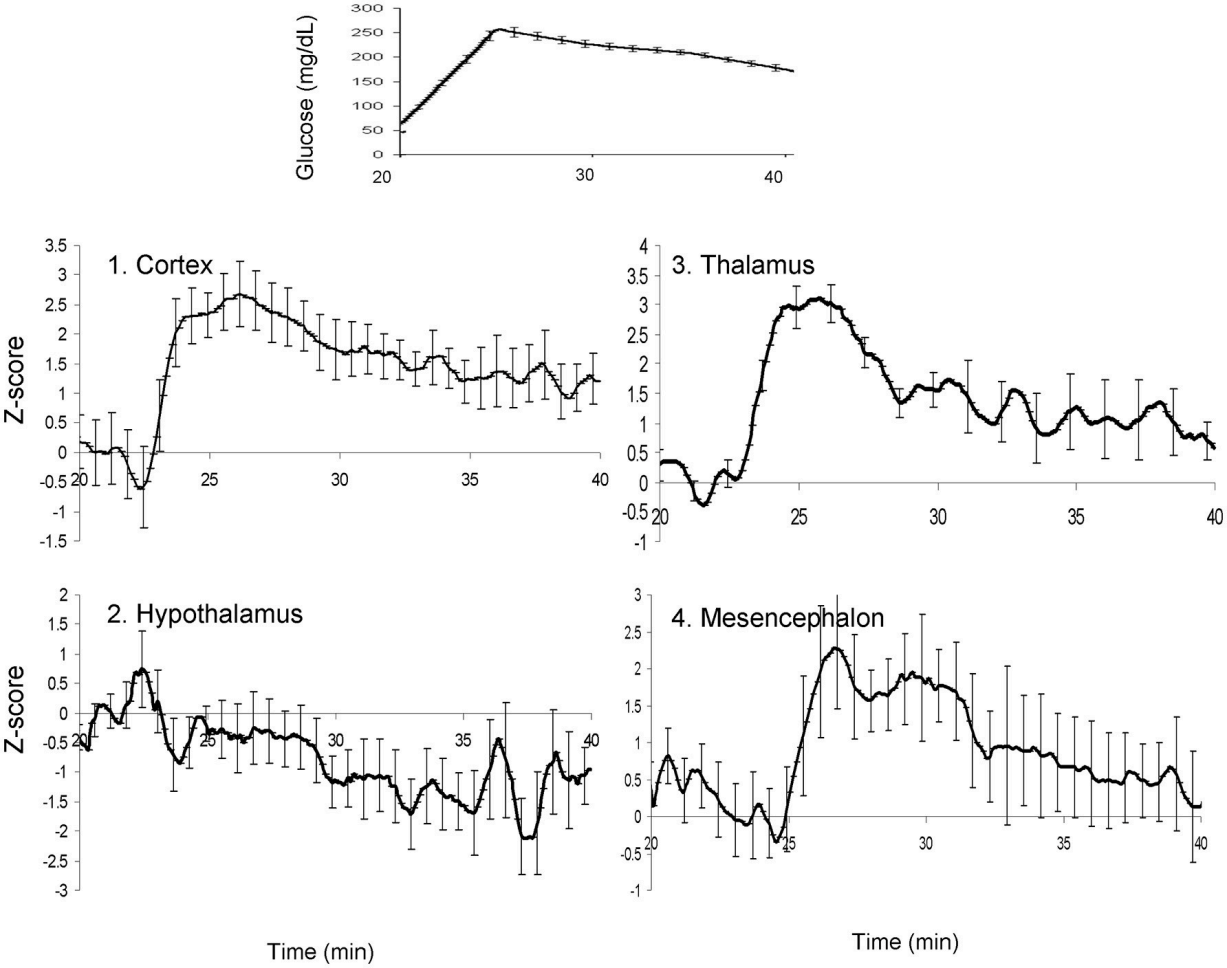
**The average time-trends for the blood glucose concentrations (top) and four cerebral regions of interest following glucose uptake**.

**Figure 6:**
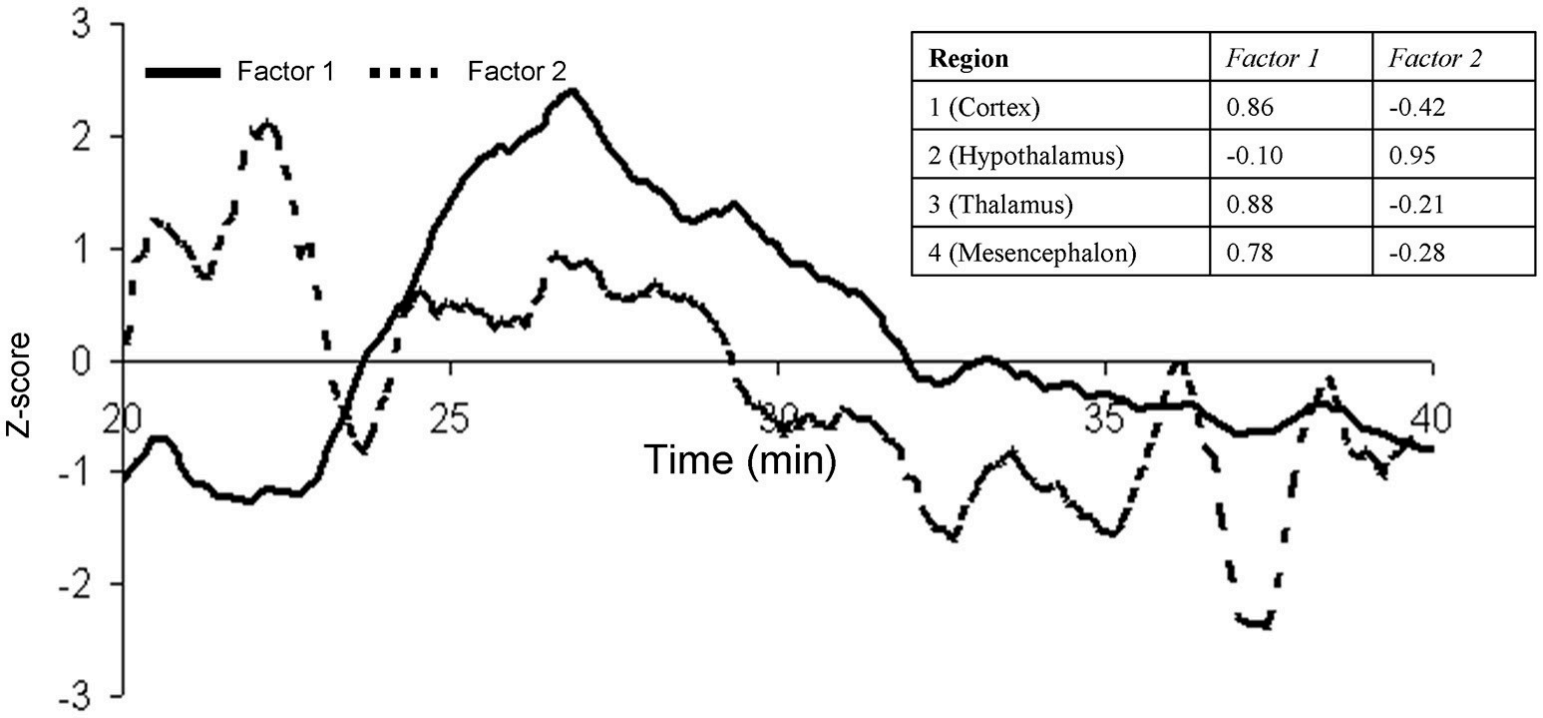
**Factor analysis was used to distill the trends the first 20 min for the four regions into two orthogonal components (Factor 1 and 2)**. Factors 1 and 2 showed a significant negative correlation (*r* = −0.72, *p* < 0.001) during the first 4 min post glucose infusion and a significant positive correlation (*r* = 0.80, *p* < 0.001) for the remaining 16 min.

Next, the regions of statistically significant CBF change were manually delineated for each animal (Figure 5). The CBF trends for each of these regions of interest were calculated and factor analysis was used to distilled their temporal trends into two orthogonal components (Factor 1 and 2), which explained 61 and 24% of the temporal variability, respectively (Figure 6). Factor 1 loaded on the CBF trends from the thalamus, cortex, and mesencephalon. Factor 2 loaded on the CBF trends from hypothalamus. The two factors, were significantly and negatively correlated (*r*^2^ = −0.72; *p* < 0.001) during the first 4 min after the glucose infusion and were significantly and positively correlated (*r*^2^ = 0.80; *p* < 0.001) thereafter (Figure 6). Finally, the MTT, time between the bolus infusion of the contrast agent and when its cerebral concentration reached maximum, was calculated to be 7.8 ± 2.1 s.

## Discussion

We combined perfusion fMRI, time-resolved sampling of glucose, insulin and C-peptide concentrations and a data-driven analysis techniques to study the pattern of CBF change during the transition from fasting glycemia to hyperglycemia. We performed this study as a part of developing a NHP model to study the physiology and genetics of human metabolic disorders. Old-world NHP, such as baboons, are likely to provide relevant scientific models because they naturally develop many metabolic diseases observed in humans and therefore offer clinically relevant responses to therapeutic interventions. We aimed to study the temporal relationship among the regional CBF trends and the regulatory response to a large change in systemic glucose concentrations and compare the results in baboons to these reported by neuroimaging studies in humans. Our study was designed to better characterize temporal hypothalamic and extra-hypothalamic CBF trends during a hyperglycemic challenge. The hyperglycemic challenge, with the fasting glycemia baseline, was chosen because it produced robust hypothalamic and extra-hypothalamic CBF changes in previous imaging studies (Matsuda et al., 1999; Liu et al., 2000). Many previous neuroimaging studies have focused on the hypothalamus, leaving the extra-hypothalamic activations unexplained (Liu et al., 2000; Page et al., 2009). We used a data-driven analysis technique (Salmeron and Stein, 2002) that did not limit our attention to any specific brain region. We identified four brain regions: cortex, thalamus, hypothalamus, and mesencephalon where the temporal CBF trends were significantly correlated with the temporal trend of the blood glucose levels. Factor analysis distilled the temporal trends for these regions into two components: Factors 1 and 2. These two components were orthogonal and contrasted the CBF trends between extra-hypothalamic (cortex, thalamus, and mesencephalon) regions (Factor 1) and the hypothalamus (Factor 2). We studied the temporal relationship among them and the time-trends of peripheral concentrations of insulin and C-peptide.

Our first aim was to replicate previous findings of hypothalamic involvement in regulation of systemic glycemia. The brain's nutrient and insulin sensing circuits converge in the hypothalamus, which regulates the systemic response to the changes in systemic glycemia levels (Lam, 2010). In agreement with prior findings, the hypothalamic CBF (factor 2) rose within 30 s following the glucose infusion and this timing was consistent with the MTT (~8 s) and the delay in the hemodynamic response (~15 s). Factor 2 remained elevated for ~4 min during which the insulin and C-peptide levels peaked and this was consistent with the typical timing of insulin release (Cook, 1983). The release of insulin is triggered by two pathways, peripherally, via the pancreatic auto-regulation, and cephalically, via the hypothalamo-pancreatic axis (Chieri et al., 1975; Moltz et al., 1975). Direct administration of glucose to the hypothalamus was shown to trigger the release of insulin (Chieri et al., 1975) and the counter-responses are activated by the direct administration of insulin (Marty et al., 2007). We consider the occurrence of the elevated hypothalamic CBF and release of insulin as a suggestive evidence for activation of the hypothalamo-pancreatic axis; however, further research will be necessary to verify this.

Our second aim was to replicate the findings of extra-hypothalamic CBF change during the hyperglycemic challenge and attempt to explain its physiology. We observed a temporal relationship between the hypothalamic and the extra-hypothalamic CBF changes. Extra-hypothalamic increase in CBF during glycemic challenge was previously reported by several studies but its physiological meaning was unclear (Page et al., 2009). The CBF change in hypothalamus and extra-hypothalamic regions followed a two-stage relationship. The first stage corresponded to the peak concentrations of insulin and C-peptide. During this stage, which took place during the first 4 min after glucose infusion, the extra-hypothalamic CBF was inversely correlated with hypothalamic CBF (*r*^2^ = −0.68, *p* < 0.001). The rise in the hypothalamic CBF corresponded to a decline in CBF elsewhere. The second stage occurred during the decline of blood glucose and plasma insulin levels. This is demonstrated by the continued, near-the-peak values of C-peptide and significant drop (by 20–30%) in insulin and glucose levels, presumably due to their clearance from the circulation by the liver and muscle tissues. Both Factor 1 and 2 rose and Factor 1 remained elevated (by about 8%) for over 10 min and the two factors were significantly and positively correlated during this time (*r*^2^ = 0.81, *p* < 0.001).

A combination of two mechanisms, the synthesis of brain's glycogen (Paulson et al., 2010) and the activity of the cerebral networks active at rest (Smith et al., 2009) can potentially explain the physiology and the spatial pattern of extra-hypothalamic CBF increases. Brain's glycogen is stored in astroglial cells and its concentrations (3–6 μmol/g) represent a significant glucose reservoir relative to free blood glucose (Oz et al., 2009). The metabolism of brain's glycogen is modulated by the ambient concentrations of glucose and insulin (Brown and Ransom, 2007). It is used as an energy substrate to meet immediate neuronal energy during activations (DiNuzzo et al., 2011) and when glucose supply from the blood is inadequate. Two recent studies demonstrated that brain's glycogen concentrations change with alteration in glycemic state. First one is a study by Choi and colleagues in rats. They demonstrated that during the transition from hypoglycemia to euglycemia brain's glycogen concentration rose until exceeding (by three- to five-fold) the pre-hypoglycemia level (Choi et al., 2003). The second one is a study by Oz and colleagues in humans, where they demonstrated that brain's glycogen concentrations declined from euglycemic baseline during modest hypoglycemia and that glycogen concentrations increased to the point of “supercompensation” upon restoration of glycemia (Oz et al., 2009). These data suggest that increase in the extra-hypothalamic blood flow is associated with synthesis of brain glycogen. Glycogen synthesis occurs in astrocytes and is associated with release of vasoactive substances (Shulman et al., 2001; Dunn and Nelson, 2010). This was shown to cause relaxation of endothelial cells of parenchymal arterioles and increase in the regional CBF (Shulman et al., 2001; Dunn and Nelson, 2010).

The regional pattern of the extra-hypothalamic CBF increase can potentially be explained by activity of the cerebral networks that are active during rest. The spatial patterns of three independent resting (default motor-and-sensory and the executive control) networks were significantly correlated and explained 26, 8, and 6% of the spatial variance in the glucose-CBF correlation patterns, respectively. Spontaneous fluctuations in resting brain give rise to low frequency (<0.1 Hz) coherent and structured functional networks (Fox and Raichle, 2007). The regional pattern of the increase in CBF during hyperglycemic challenge showed the largest spatial overlap with the default mode network. The default-mode characterizes basal neural activity and non-directed cognitive processing and diminishes during goal-directed activity (Fox and Raichle, 2007). This network is intrinsic to the primate brain and is readily observed in sleeping infants (Fransson et al., 2007) and in anesthetized NHPs (Vincent et al., 2007). The high spatial correlation between the SPM of CBF increases and the default mode network was driven by the overlap in the spatial patterns that included partial lobe, thalamus, and the prefrontal and cingulate cortices. The mesencephalon and thalamus are also frequently observed in the same independent network components (Biswal et al., 2010). Two other networks that showed significant spatial overlap, specifically in the frontal and parietal areas, included the motor-and-sensory and the executive networks. Our data suggest that the extra-hypothalamic CBF could be associated with uptake of glucose and increases in brain's glycogen supply, specifically in the nodes of the brain networks that are active in the anesthetized state. This is consistent with previous findings, in which cerebral glucose metabolism was demonstrated to be highly associated with brain activity at varying doses of anesthesia (Shulman et al., 2009). The significant spatial overlaps between glucose metabolism-induced CBF increases and the default mode network may thus serve as an index for brain energy-activity interaction during hyperglycemia. However, the highest spatial correlation, with the default mode network, explained only 26% of the variance and therefore the role of cerebral networks in explaining the spatial pattern of extra-hypothalamic CBF changes that are active during rest remains putative. Additionally, the potential role of glycogen as a transferable metabolic intermediates has been questioned based on its high-energetic cost, suggesting that the energetic dependence of neurons on astrocytic glycogen is modest (DiNuzzo et al., 2011). Further investigation that includes spectroscopic and/or FDG-PET based assessments of brain's glucose metabolism rate and glycogen concentrations are necessary to get a better understanding of this physiological mechanism.

## Limitations

Anesthetic drugs such as s-ketamine and isoflurane are known to act as vasodilators and can increase CBF independently of the glucose and oxygen metabolism levels (Långsjö et al., 2005). Ketamine sedation was shown to reduce glucose clearance, insulin secretion and counter regulatory hormonal production in a NHP model (Lehmann et al., 1997) and 2% isoflurane altered glucose uptake in the brain in a rodent model (Nasrallah et al., 2013). While, in our previous investigation of the effects of ketamine anesthesia on CBF in healthy baboons, we observed only minor regional CBF changes in response to different levels of ketamine anesthesia (dose ranging from 4.8 to 14.6 mg/kg; Szabó et al., 2008), the effects of anesthetics on the CBF response to glucose infusion cannot be ruled out. However, the use of anesthesia (including isoflurane) in whole body metabolic studies using several NHP models, including baboons, is accepted practice and produces robust clinically translational data (Bodkin et al., 1989; Chavez et al., 2008; Wang et al., 2013; Tozzo et al., 2015), although some studies have described alterations under certain conditions in some animal species (Laber-Laird et al., 1992). Nevertheless, it is unlikely that the necessity of ketamine/isoflurane use in baboons precludes its utility in whole body metabolic disease studies in controlled experimental settings.

Another potential limitation of this study is the difference in animal handling protocols between the hypoglycemic challenges and the resting-state experiments. Vecuronium bromide was used in the resting-state experiment to simplify the placement of the tracheal tube. Vecuronium bromide can suppress the central respiratory activity and this can potentially alter the spatial patterns of resting state cerebral networks. We believe this is a minor limitation because vecuronium bromide was not used beyond initial dose and because animals were mechanically ventilated at the same rate in both experiments.

In addition, further research is needed to clarify whether the CBF changes are reduced if euglycemic or mild hyperglycemic baseline is used during the hyperglycemic challenge. FDG-PET studies report that the regional cerebral uptake values are inversely correlated with the baseline plasma glucose concentrations (Kawasaki et al., 2008) and this suggests that a similar trend could be observed for the regional CBF changes. This analysis did not account for partial volume effects. However, the high-resolution pCASL imaging (2 × 2 × 3 mm) protocol produced no apparent partial volume artifact in functional experiments in NHP. The phase errors introduced by imperfect labeling pulses could be potential confounders affecting the absolute CBF quantification. Although, our study aims to compare percent CBF differences before and after glucose administration, future studies implementing a multi-phase pCASL (Jung et al., 2010) technique could further improve the accuracy of the results.

## Conclusions

Our results support the use of the baboon as a pertinent nonhuman primate model and neuroimaging techniques to study changes in cerebral physiology during metabolic challenges. Our study in baboons complemented previously reported findings with time-resolved measurements of cerebral perfusion associated with changing blood glucose and plasma insulin levels. We observed that changes in the hypothalamic CBF occurred concomitantly with the regulatory response to a systemic glucose change and can potentially be explained by the activation of the hypothalamo-pancreatic axis. The physiology and the spatial pattern of extra-hypothalamic CBF can putatively be explained by the glycogen shunt and the activity of the default mode network. Further investigations that include assessments of brain's glucose metabolism rate and glycogen concentrations are necessary to get a better understanding of the putative role of these physiological mechanisms.

## Author contributions

PK and VV conceived and designed the study; PK, HW, and VV analyzed the data and wrote the manuscript. HW, MD, DW, and AL provided advice, analysis tools, and MRI pulse sequences. PK, VV, PH, RB, MA, and VM participated in the data collection and biochemical analyses. Manuscript edits were performed by JL, HW, MD, DW, AL, RB, PF, PH, PTF, AC, and VV. Additional consultation on the study design and data collection was provided by PTF and AC. All authors have read and approved the final manuscript.

## Funding

This research was primarily supported by the Max and Minnie Tomerlin Voelcker Foundation, a IIMS/CTSA Translational Technologies and Resources Supplemental Award, and P30 DK 056350 Nutrition Obesity Research Center grant (Nutrigenetics/Nutrigenomics core) to VV. HW is supported by a Predoctoral Fellowship (11PRE5670005) from the American Heart Association. This research was conducted using facilities constructed with support from the Research Facilities Improvement Program under grant number C06 RR (numbers 015456, 013556, 017515) from the National Center for Research Resources and with support from National Institute for Health grant P51 RR013986.

### Conflict of interest statement

The authors declare that the research was conducted in the absence of any commercial or financial relationships that could be construed as a potential conflict of interest.
